# Supplementary material for: Doxycycline induces Hok toxin killing in host E. coli
Source: PLoS One. 2020 Jul 6;15(7):e0235633. doi: 10.1371/journal.pone.0235633 (PMC7337300; doi:10.1371/journal.pone.0235633)
Supplement: Attachment — Submitted filename: Reviewers recommendations.docx [file pone.0235633.s001.docx]

**DOXYCYCLINE 1 INDUCES HOK TOXIN KILLING IN HOST E. COLI**

**Reviewer's recommendations**

Dear authors

In general it is an interesting article and the changes are minor

Line 100 correct write CaCl2

Line 142 to line 147, all this information has already been presented in the introduction and in the methodology, so it is recommended to go to the results directly

Line 216 the name of the medium used capitalize it LB and LB amp

Line 231 and 232 the names of the media used must be capitalized LB LB amp

Table 1. The names of the media used must be capitalized LB and LB amp

It is recommended to improve the presentation of figure 1 and avoid that the arrows are above the text

Figure 8 is pixelated, improve the quality of it
