# Supplementary material for: Doxycycline induces Hok toxin killing in host E. coli
Source: PLoS One. 2020 Jul 6;15(7):e0235633. doi: 10.1371/journal.pone.0235633 (PMC7337300; doi:10.1371/journal.pone.0235633)
Supplement: Attachment — Submitted filename: DOXYCYCLINE INDUCES HOK TOXIN KILLING IN HOST E. COLI.docx [file pone.0235633.s002.docx]

DOXYCYCLINE INDUCES HOK TOXIN KILLING IN HOST E. COLI

Chukwudi et al

The manuscript provides preliminary evidence supporting the hypothesis that doxycycline inhibition of the bacterial hok/sok dsRNA complex degradation by RNAse III consequently enhances the killing effect of hok. Furthermore, this effect of doxycycline appears independent of other antibiotics and could be exploited to provide synergistic and more effective outcomes in treatment of antibiotic resistance mediated via ESBL-gene harboring plasmids.

The work has a logical hypothesis founded on substantial preexisting work in the field, and the manuscript reads reasonably well. However, two major caveats detract from the manuscript. Firstly, the relevant results involve SOS deficient E.coli lab strains where the effects are most pronounced, but how these extrapolate to wild type is not discussed; and secondly, it is assumed all along that all observed growth effects are caused in a dose dependent manner by the targeted action of doxycycline on RNAse III as opposed to other non-targeted effects.

I recommend that the manuscript be re-written resubmitted to include the discussion addressing these major caveats.

There are other instances where changes are required

- Presenting and tabulating differences in growth in a quantitative manner (rate of growth)
- Line:140

It is not clear what experiment the section starting at Line 140 refers to or, what “both plasmids” in line 146 relate too.

- Line 182-186

The description here is unnecessarily long and needs to be rephrased for clarity. Figure 3 shows the dose dependent inhibition of a “rescue” phenotype in hok/sok+ SOS- bacteria. It is assumed in the manuscript that this is a direct result of the doxycycline inhibition of RNASe III but is not proven. This has to be clearly indicated in a discussion and mentioning alternative explanations along with arguments against them.

Some minor points

- 178: Suggest starting with “While 1uM completely inhibited hok/sok+……”
- 170: Sentence in line 170 needs references.
- 172: The name of the SOS strain needs to indicated here
- 202:   In *the* Top 10 strain,
- 205:  Rewrite to “….compared in LB media with and without ampicillin”
