# Supplementary material for: Doxycycline induces Hok toxin killing in host E. coli
Source: PLoS One. 2020 Jul 6;15(7):e0235633. doi: 10.1371/journal.pone.0235633 (PMC7337300; doi:10.1371/journal.pone.0235633)
Supplement: Attachment — Submitted filename: Response to reviewers2.docx [file pone.0235633.s003.docx]

**DOXYCYCLINE 1 INDUCES HOK TOXIN KILLING IN HOST E. COLI**

**Reviewer's recommendations**

Dear authors

In general it is an interesting article and the changes are minor

Line 100 correct write CaCl2-Cacl corrected to CaCl

Line 142 to line 147, all this information has already been presented in the introduction and in the methodology, so it is recommended to go to the results directly-lines 142-145 deleted. Opening sentence modified.

Line 216 the name of the medium used capitalize it LB and LB amp-done

Line 231 and 232 the names of the media used must be capitalized LB LB amp- done

Table 1. The names of the media used must be capitalized LB and LB amp- done

It is recommended to improve the presentation of figure 1 and avoid that the arrows are above the text-both presentation and quality improved.

Figure 8 is pixelated, improve the quality of it-Fig 8 quality improved.

Reviewer #2: The manuscript provides preliminary evidence supporting the hypothesis that doxycycline inhibition of the bacterial hok/sok dsRNA complex degradation by RNAse III consequently enhances the killing effect of hok. Furthermore, this effect of doxycycline appears independent of other antibiotics and could be exploited to provide synergistic and more effective outcomes in treatment of antibiotic resistance mediated via ESBL-gene harboring plasmids.
The work has a logical hypothesis founded on substantial preexisting work in the field, and the manuscript reads reasonably well. However, three major caveats detract from the manuscript. Firstly, the relevant results involve SOS deficient E.coli lab strains where the effects are most pronounced, but how these extrapolate to wild type is not discussed; The SOS deficient strain (SS996) was used to compare the effects observed in the normal lab strain (Top 10) because the *hok/sok* locus is known to function as a stress response element in *E. coli* , and complements bacterial SOS response. The discussion has been modified to more clearly explain the rational for using this strain. The following text has been added: “The observed increase in doxycycline susceptibility in this study was found to be more prominent in the SOS-deficient strain (SS996) than in the normal strain (Top 10). This is consistent with previous reports that the growth effects of the *hok/sok* locus is more prominent in strains that are defective in the SOS response (16), and has been associated with alterations in FtsZ activity (27). The SOS response pathway has been implicated in bacterial persistence to β-lactam antibiotics and the fluoroquinolones, but conversely increases doxycycline susceptibility in this study.” Also, with the discussion section, we explain that the effects will be examined in a range of clinical isolates in future.

and secondly, it is assumed all along that all observed growth effects are caused in a dose dependent manner by the targeted action of doxycycline on RNAse III as opposed to other non-targeted effects. This is a helpful comment. The inhibitory effects of antibiotics are often complex, and the growth effects observed in this study reflect both inhibition of RNAase III as well as other targets of tetracycline. We have extensively modified the manuscript with the addition of a section of analysing the growth effects of doxycycline in the light of other known inhibitory mechanisms. Here, we have demonstrated increased doxycycline susceptibility in the presence of the *hok/sok* locus, even though there is some level of susceptibility without the *hok/sok* (as would be expected of any antibiotics). To explain the situation more clearly, we have inserted additional text within the discussion section to explain that multiple antimicrobial mechanisms are possible, thus: “The locus increases both the lag time and exponential growth rates of host bacteria cultures. In this study, we show that whereas the *hok/sok* will generally increase doubling time and average growth rate of host bacteria cultures, it inadvertently confers increased doxycycline susceptibility to host cells especially at very low doses that the cells would otherwise not have been susceptible. This increased susceptibility to doxycycline appear to be mediated via an additional mechanism that acts synergistically with its established antibacterial mechanism of protein synthesis inhibition. This is particularly interesting, considering the fact that the *hok/sok* locus enhances ampicillin (and β-lactam antibiotics) resistance in host bacteria cells (16).”

Third, the manuscript lacks any statistical inferences. We have added inferential statistical analyses in the Data Analysis subsection of Materials and Methods. In the result section, we have added a new segment on “**Effect of doxycycline on the growth curve characteristics of hok/sok host E. coli.**” with p-values, which was also added to the segment on “**Effects of doxycycline on the morphology of *hok/sok* host cells”.**

I recommend that the manuscript be re-written resubmitted to include the discussion addressing these major caveats.

There are other instances where changes are required

• Presenting and tabulating differences in growth in a quantitative manner (rate of growth) with significance values. The rate of growth has now been calculated and presented clearly in table 2, and significance values included in the text..

• Line:140
It is not clear what experiment the section starting at Line 140 refers to or, what “both plasmids” in line 146 relate too.- the plasmid identities have been clarified: the text now reads “…. *E. coli* Top 10 strain, in which both *hok/sok^+^* and control plasmids, pCCB1 and pUC19 are very stable without antibiotic selection)….”

• Line 182-186
The description here is unnecessarily long and needs to be rephrased for clarity. –Rephrased to now read “This suggests that the growth enhancing effects of the *hok*/*sok* locus also increases its susceptibility to doxycycline. In other words, the more the *hok*/*sok* locus enhances bacterial stress response and survivability, the more it increases the host cell susceptibility to doxycycline.”

Figure 3 shows the dose dependent inhibition of a “rescue” phenotype in hok/sok+ SOS- bacteria. It is assumed in the manuscript that this is a direct result of the doxycycline inhibition of RNASe III but is not proven. This has to be clearly indicated in a discussion and mentioning alternative explanations along with arguments against them. The results and discussion sections have been modified to include these considerations, as indicated above.

Some minor points

• 178: Suggest starting with “While 1uM completely inhibited hok/sok+……”-sentence modified, now reads “While 1 µM doxycycline completely inhibited the growth of the *hok/sok*^+^ cells, the *hok/sok*^-^ cells was not inhibited to even half the normal level at the same drug concentration”

• 170: Sentence in line 170 needs references. Reference inserted

• 172: The name of the SOS strain needs to indicated here-indicated (SS996).

• 202: In the Top 10 strain,-done
• 205: Rewrite to “….compared in LB media with and without ampicillin”-done.

Journal Requirements:

When submitting your revision, we need you to address these additional requirements.

1. Please ensure that your manuscript meets PLOS ONE's style requirements, including those for file naming. Checked and OK.

2. Please include a copy of Table 2 which you refer to in your text on page 11.-Table included (changed to Table 3).

Please include the following items when submitting your revised manuscript:

- A rebuttal letter that responds to each point raised by the academic editor and reviewer(s). This letter should be uploaded as separate file and labeled 'Response to Reviewers'.
- A marked-up copy of your manuscript that highlights changes made to the original version. This file should be uploaded as separate file and labeled 'Revised Manuscript with Track Changes'.
- An unmarked version of your revised paper without tracked changes. This file should be uploaded as separate file and labeled 'Manuscript'.
